# Gene Expression and DNA Methylation Alterations During Non-alcoholic Steatohepatitis-Associated Liver Carcinogenesis

**DOI:** 10.3389/fgene.2019.00486

**Published:** 2019-05-29

**Authors:** Kostiantyn Dreval, Volodymyr Tryndyak, Aline de Conti, Frederick A. Beland, Igor P. Pogribny

**Affiliations:** ^1^Division of Biochemical Toxicology, National Center for Toxicological Research, Jefferson, AR, United States; ^2^Program in Cancer Genetics, Epigenetics and Genomics, Division of Molecular Medicine, Department of Internal Medicine, University of New Mexico Comprehensive Cancer Center, Albuquerque, NM, United States

**Keywords:** non-alcoholic steatohepatitis, hepatocellular carcinoma, gene specific methylation, H3K4me3, *Tubb2b*

## Abstract

Hepatocellular carcinoma (HCC) is one of the most aggressive human cancers. HCC is characterized by an acquisition of multiple abnormal phenotypes driven by genetic and epigenetic alterations, especially abnormal DNA methylation. Most of the existing clinical and experimental reports provide only a snapshot of abnormal DNA methylation patterns in HCC rather than their dynamic changes. This makes it difficult to elucidate the significance of these changes in the development of HCC. In the present study, we investigated hepatic gene expression and gene-specific DNA methylation alterations in mice using the Stelic Animal Model (STAM) of non-alcoholic steatohepatitis (NASH)-derived liver carcinogenesis. Analysis of the DNA methylation status in aberrantly expressed epigenetically regulated genes showed the accumulation of DNA methylation abnormalities during the development of HCC, with the greatest number of aberrantly methylated genes being found in full-fledged HCC. Among these genes, only one gene, tubulin, beta 2B class IIB (*Tubb2b*), was increasingly hypomethylated and over-expressed during the progression of the carcinogenic process. Furthermore, the *TUBB2B* gene was also over-expressed and hypomethylated in poorly differentiated human HepG2 cells as compared to well-differentiated HepaRG cells. The results of this study indicate that unique gene-expression alterations mediated by aberrant DNA methylation of selective genes may contribute to the development of HCC and may have diagnostic value as the disease-specific indicator.

## Introduction

Hepatocellular carcinoma (HCC), which accounts for almost 90% of all primary liver malignancies, is one of the most aggressive and enigmatic human cancers with steadily increasing incidence in the United States and worldwide (Llovet et al., 2016; Bertuccio et al., 2017). The development of HCC is associated with well-identified etiological risk factors, including chronic hepatitis B (HBV) and C (HCV) viral infections, chemical exposure, and excessive alcohol consumption (Llovet et al., 2016); however, the contribution of specific risk factors to HCC development varies greatly by geographic location. Currently, chronic HBV infection and exposure to the fungal metabolite aflatoxin B_1_ are the predominant risk factors for HCC in Southeast Asia and Africa, whereas chronic HCV infection is the main HCC risk factor in the United States and Western countries (Choo et al., 2016). The contribution of specific etiological factors to the development of HCC is dynamic and expected to change due to recent progress in the primary prevention of HCC induced by HBV and HCV infection, the arrival of a new generation of direct HCV antiviral drugs, and the fast-rising incidence of NAFLD. Recent evidence indicates that non-alcoholic steatohepatitis (NASH) is becoming a prevalent risk factor of HCC, replacing viral hepatitis and alcohol-related liver diseases as the major etiological cause of HCC (Younossi et al., 2015; Marengo et al., 2016; Younes and Bugianesi, 2018; Kim et al., 2019).

In addition to lifestyle and environmental risk factors, HCC is a disease characterized by the presence of multiple heritable abnormal cellular phenotypes driven by genetic (Zucman-Rossi et al., 2015; The Cancer Genome Atlas Research Network, 2017) and epigenetic alterations (Pogribny and Rusyn, 2014; The Cancer Genome Atlas Research Network, 2017). While the role of genetic abnormalities and sequential progression of pathomorphological lesions in liver carcinogenesis are well-characterized, the underlying epigenetic mechanisms, in general, and cancer-related cytosine DNA methylation aberrations, in particular, in the development of HCC are still poorly understood and require special attention. The primary function of cytosine DNA methylation is to defend the genome by controlling the accurate expression of genetic information (Jones, 2012; Ehrlich and Lacey, 2013; Edwards et al., 2017). Cytosine DNA methylation, mainly but not exclusively, functions as a transcriptional “ON-OFF” switch at regulatory regions: the occurrence of DNA methylation at unmethylated CpG sites inhibits transcription, whereas demethylation of methylated CpG sequences activates transcription (Bestor et al., 2015).

Numerous studies have documented profound gene-specific DNA methylation aberrations in key cancer-related pathways in full-fledged HCC (Villanueva et al., 2015; Yamada et al., 2016; Zhang et al., 2016) as well as in preneoplastic lesions (Murphy et al., 2013); however, the majority of the existing reports provide only a snapshot of DNA methylation abnormalities in HCC rather than their dynamic changes. This makes it difficult to clarify the role and functional significance of DNA methylation alterations in the development of HCC. Additionally, while DNA methylation as a mechanism of controlling gene transcription has been studied extensively, the longstanding question of the causality or consequentiality of the concordant gene expression/methylation alterations in carcinogenesis, in general (Baylin and Bestor, 2002; Long et al., 2017), and liver carcinogenesis remains unresolved. Based on these considerations, in this study we investigated the role of gene-specific epigenetic and gene expression alterations in the development of HCC associated with NAFLD using STAM non-alcoholic (NASH)-derived liver carcinogenesis, a model that resembles the development of NASH-associated HCC in humans (Fujii et al., 2013; Takakura et al., 2014).

## Materials and Methods

### Animals, Experimental Design, and Treatments

In the present study, we used liver tissue samples from male mice subjected to NASH-associated hepatocarcinogenesis. This is the first mouse model that mimics the development of HCC in diabetes-associated NASH patients (Fujii et al., 2013; Takakura et al., 2014). The complete experimental design and pathomorphological description of the STAM NASH-related hepatocarcinogenesis model have been described previously by Fujii et al. (2013). Briefly, 2-day-old male C57BL/6J mice were injected with streptozotocin (200 μg/mouse) and were continuously fed a high-fat diet (CLEA Japan, Tokyo, Japan) starting from 4 weeks of age throughout the duration of the study. Control male mice were not injected with streptozotocin and were maintained on standard animal chow for the duration of the study. Liver tissue samples of STAM mice at non-alcoholic fatty liver (NAFL; 6 weeks), NASH-fibrotic (12 weeks), and full-fledged HCC (20 weeks) stages of hepatocarcinogenesis and liver samples from age-matched control mice were purchased from the SMC Laboratories., Inc. (Tokyo, Japan). All experimental procedures were performed according to the Japanese Pharmacological Society Guidelines and experimental protocols were approved by the SMC Laboratories, Inc. Research Animal Care and Use Committee.

### Cells and Cell Culture

The human progenitor hepatic HepaRG cell line was obtained from the Biopredic International (Overland Park, KS, United States) and human HCC HepG2 cell line was obtained from the American Type Culture Collection (ATCC, Manassas, VA, United States). The cells were maintained per the manufacturer’s recommendations.

### *In vitro* Oleic Acid-Induced Model of NAFL

On the 28th day after the initial seeding, the fully differentiated HepaRG cells were continuously treated with 250 μM oleic acid (Sigma-Aldrich, St. Louis, MO, United States) in the differentiation media for an additional 14 days to induce accumulation of fatty acids (Rogue et al., 2014). The cells were then harvested by mild trypsinization, washed in PBS, and frozen immediately at −80°C for subsequent analyses.

### Determination of Triglycerides Accumulation in HepaRG Cells

Triglyceride accumulation in HepaRG cells after oleic acid treatment was quantified using AdipoRed^TM^ Assay Reagent (Lonza, Walkersville, MD, United States). Briefly, the cells were washed in PBS and AdipoRed^TM^ Assay Reagent was added. After a 10-min incubation at room temperature, the fluorescence intensity was quantified using a Synergy^TM^ H4 hybrid multi-mode microplate reader (BioTek, Winooski, VT, United States).

### Total RNA Isolation and qRT-PCR

Total RNA was extracted from liver tissue samples of male STAM mice (*n* = 4/group/treatment) using miRNeasy Mini kits (Qiagen, Valencia, CA, United States). Total RNA (2 μg) was reverse transcribed using random primers and High Capacity cDNA Reverse Transcription kits (Life Technologies, Grand Island, NY, United States), and gene expression was determined by quantitative reverse-transcription PCR (qRT-PCR) using TaqMan gene expression assays and the primers listed in the Supplementary Table 1. TATA box binding protein (*Tbp*) was used as an endogenous control. The relative amount of each mRNA transcript was determined using the 2^−ΔΔCt^ method (Schmittgen and Livak, 2008).

### Methylated DNA Immunoprecipitation (MeDIP)-Quantitative PCR Analysis

Genomic DNA was isolated from mouse liver tissues of control and STAM mice using DNeasy Blood and Tissue kits (Qiagen). MeDIP was performed with MethylMiner Methylated DNA Enrichment kits (Invitrogen, Carlsbad, CA, United States). Briefly, 1 μg of genomic DNA was isolated from the NAFL (6 weeks), NASH-fibrotic (12 weeks), and full-fledged HCC (20 weeks) tissue samples and from liver tissue samples of corresponding age-matched control mice. The DNA samples were randomly sheared by sonication to an average range of 0.2–1.0 kb. Ninety percent of the sheared DNA was incubated overnight at 4°C with MBD-Biotin protein coupled to M280 Streptavidin Dynabeads. The remaining 10% of the sheared DNA (“input DNA”) was used to quantify the amount of DNA used for the MeDIP analysis. The captured methylated DNA was eluted as a single fraction using a high-salt elution buffer and purified by ethanol precipitation. The methylation status of the CGIs located within the 5′-UTR/first exon region of selected genes was determined by qPCR of DNA from immunoprecipitated and unbound DNA using primer sets listed in Supplementary Table 1. The results were normalized to the amount of input DNA. The levels of *Gapdh* gene promoter methylation and IAP repetitive element methylation were assessed for the assay performance control (Supplementary Figure 1A).

### MeDIP-Microarray Analysis

Methylated DNA immunoprecipitation was performed with MagMeDIP kits (Diagenode, Denville, NJ, United States) using 1 μg of genomic DNA isolated from full-fledged HCC (20 weeks) tissue samples and from liver tissue samples of corresponding age-matched control mice. The immunoprecipitated DNA and input DNA pellets were labeled with cyanine 5-dUTP and cyanine 3-dUTP, respectively, using Agilent SureTag DNA Labeling kits (Agilent Technologies, Santa Clara, CA, United States). The data acquisition and analysis was performed as described in Tryndyak et al. (2016). A list of differentially methylated CGIs was generated by calculation of Benjamini–Hochberg adjusted *p*-values (Benjamini and Hochberg, 1995) to control the FDR in multiple testing data, with an adjusted *p*-value cut-off 0.05, and a *Z*-score fold-change threshold of 1.5.

### Reduced Representation Bisulfite Sequencing (RRBS) Analysis of Genome-Wide DNA Methylation

Genomic DNA from HepaRG and HepG2 cells was isolated with DNeasy Blood and Tissue Kit (Qiagen) and RRBS libraries were prepared with NEBNext^^®^^ Ultra^TM^ DNA Library Prep Kit for Illumina^^®^^ (New England Biolabs, Ipswich, MA, United States) per the manufacturer’s protocol. The RRBS library preparation, next-generation sequencing, data analysis, and digital methylation quantitation were performed as described in Tryndyak et al. (2017).

### Chromatin Immunoprecipitation Assay

Formaldehyde cross-linking and the ChIP assay, with primary antibody against trimethylated histone H3 lysine 4 (Abcam, Cambridge, MA, United States), were performed using Magna ChIP^TM^ A – Chromatin Immunoprecipitation kits (Millipore Corporation, Burlington, MA, United States). Purified DNA from immunoprecipitated and input DNA was analyzed by qPCR with the same primers used in the MeDIP assay (Supplementary Table 1). The results were normalized to the amount of input DNA and presented as fold change calculated from the difference between the DNA from livers of mice from the experimental groups relative to that in control mice. The levels of H3K4me3 enrichment in the gene desert region of chromosome 6 (Mouse Negative Control Primer Set 1; Active Motif, Carlsbad, CA, United States) and in the promoter region of *Gapdh* gene were assessed for the assay performance control (Supplementary Figure 1B).

### Retrieval of Gene Expression Data From Online Databases

The high-throughput whole genome microarray analyses and the gene expression profiles in the livers of control mice and mice subjected to the STAM model of liver carcinogenesis are detailed in de Conti et al. (2017) (NCBI’s GEO database; accession number GSE83596). The gene expression data in HepaRG and HepG2 cells were downloaded from the publicly available GEO dataset (accession number GSE40117). The gene expression in human HCC and tumor pathological data were extracted as .txt files from The Cancer Genome Atlas database (TCGA^1^). The RSEM software package was used for TCGA RNA-Seq gene expression quantitation and normalization (Li and Dewey, 2011). RSEM-normalized expression values were used for differential gene expression analysis.

### Statistical Analyses

Results are presented as mean ± SD. Student’s *t*-test was used to evaluate significant differences between STAM mice and age-matched control mice at the same time point. One-way analysis of variance (ANOVA), followed by Tukey *post hoc* analysis, was used to evaluate significant differences between the stages during the progression to HCC. When necessary, the data were natural log transformed before conducting the analyses to maintain an equal variance or normal data distribution. Simple linear regression was applied to calculate the trends. Pearson product-moment correlation coefficients were used to determine the relationship between gene expression and gene-specific methylation or level of histone modification. Values of *P* < 0.05 were considered significant.

## Results

### Methylation Status of Common Differentially Expressed Genes in the Livers of STAM Mice

Previously, we demonstrated that the development of NASH-derived HCC in male STAM mice was characterized by substantial stage-dependent alterations in gene expression as compared to the age-matched control mice (de Conti et al., 2017). Among differentially expressed genes (a twofold change in gene expression and Benjamini-Hochberg adjusted *p*-value <0.05), 60 genes exhibited the same trend of the expression changes at each stage of NASH-associated liver carcinogenesis, at NAFL (6 weeks), NASH-fibrotic (12 weeks), and full-fledged HCC (20 weeks) stages. Analysis of the promoter region of these 60 genes identified 35 CpG island-containing genes that could be epigenetically regulated. This was indicated by the presence of a strong CpG island in the gene promoter regions, based on well-established criteria: greater than 500 bp in length, G+C greater than 55%, and an observed CpG/expected CpG ratio of >0.65 (Takai and Jones, 2002).

**FIGURE 1:**
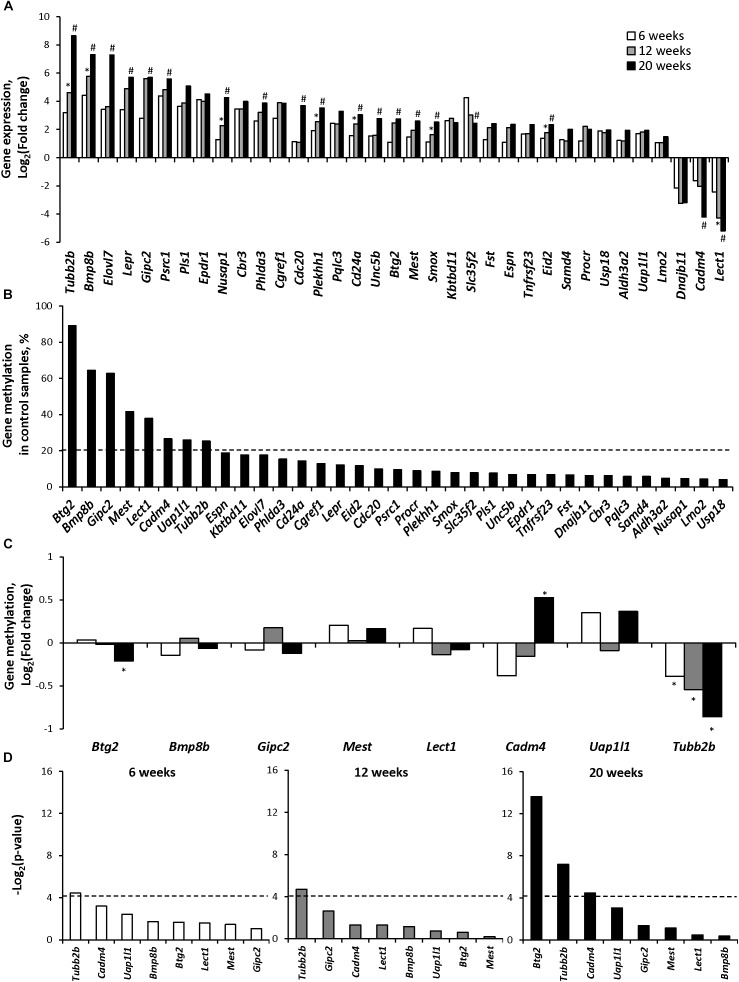
Gene expression and gene-specific methylation of epigenetically regulated differentially expressed genes during NASH-associated liver carcinogenesis in STAM mice. **(A)** Expression of the selected epigenetically regulated genes at NAFL (6 weeks), NASH-fibrotic (12 weeks), and full-fledged HCC (20 weeks) stages of NASH-associated liver carcinogenesis. ^∗^Denotes significantly expressed genes (*P* < 0.05) in the livers of STAM mice at 12 weeks as compared to their expression at 6 weeks. ^#^Denotes significantly expressed genes (*P* < 0.05) in the livers of STAM mice at 20 weeks as compared to their expression at 6 and 12 weeks. **(B)** Gene-specific methylation of selected genes in the livers of control mice. Dashed line indicates a 20% threshold methylation level. **(C)** Gene-specific DNA methylation changes in the promoter region of the upregulated genes that passed 20% threshold DNA methylation level in control livers or genes that were down-regulated during the development of HCC. ^∗^Denotes statistically a significant (*P* < 0.05) difference in the promoter region DNA methylation in the livers of STAM mice as compared to that in the livers of age-matched control mice. **(D)** Pearson correlation *P*-value between gene expression and promoter DNA methylation changes in the livers of mice subjected to STAM hepatocarcinogenesis at 6, 12, and 20 weeks. Dashed line indicates the threshold level, which was selected as *P* = 0.05 (Log_2_ *P* = −4.13).

Figure 1A shows that in the livers of STAM mice, 32 epigenetically regulated genes were over-expressed and 3 were down-regulated as compared to the age-matched control mice. Among these genes the expression of the *Tubb2b*, *Bmp8b*, *Nusap1*, *Plekhh1*, *Cd24a*, *Smox*, *Eid2*, and *Lect1* genes was altered in a stage-dependent manner.

To determine the role of epigenetic mechanisms in the altered expression of these genes, the status of the promoter region cytosine DNA methylation was investigated. Figure 1B shows that the extent of promoter methylation in these 35 genes varied greatly in the livers of control mice ranging from 4 to 90%. To evaluate the functional significance of the DNA methylation changes, a threshold level of ≥20% cytosine methylation was applied. Based on this criterion, eight genes, *Btg2*, *Bmp8b*, *Gipc2*, *Mest*, *Lect1*, *Cadm4*, *Uap1/1*, and *Tubb2*, were detected, and the status of their gene-specific cytosine DNA methylation was investigated (Figure 1C). Among these genes, only one, *Tubb2b*, an important microtubule cytoskeleton gene (Gadadhar et al., 2017), was differentially methylated (*P* < 0.05) in NAFL, NASH-fibrotic, and HCC tissue samples as compared to that in control mice, exhibiting a 23, 31, and 45% decrease in the promoter region methylation, respectively (Figure 1C and Supplementary Table 2). A correlation analysis between gene expression and gene-specific methylation revealed that the methylation status of only the *Tubb2b* gene was inversely correlated (*P* < 0.05) with its expression at each stage of liver carcinogenesis (Figure 1D).

### Status of Gene-Specific Histone H3K4me3 in NASH-Related Hepatocarcinogenesis

**FIGURE 2:**
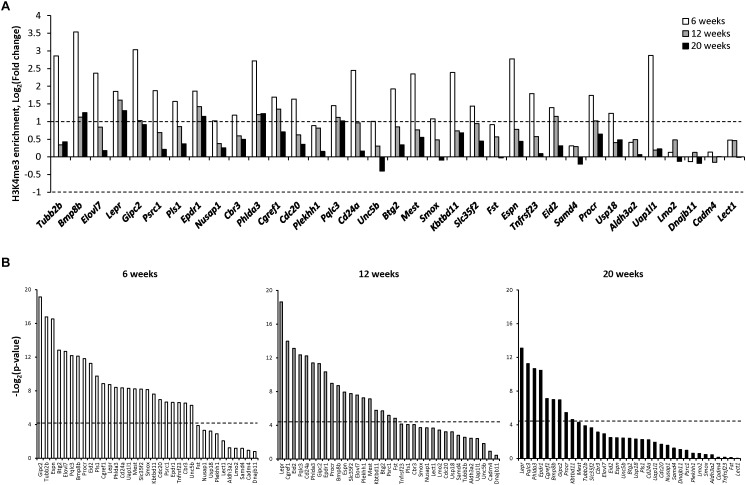
Gene-specific histone H3K4 trimethylation in the livers of STAM mice during hepatocarcinogenesis. **(A)** The changes in the level of histone H3 lysine 4 trimethylation in the promoter region of selected genes at NAFL (6 weeks), NASH-fibrotic (12 weeks), and full-fledged HCC (20 weeks) stages of NASH-associated liver carcinogenesis. Dashed line indicates threshold level, which was selected as a twofold change comparing to control age-matched mice (Log_2_ fold change = +1 and −1). **(B)** Pearson correlation *P*-value between gene expression and change in the promoter region histone H3 lysine 4 trimethylation in the livers of mice subjected to STAM hepatocarcinogenesis at 6, 12, and 20 weeks. Dashed line indicates the threshold level, which was selected as *P* = 0.05 (Log_2_ *P* = −4.13).

To elucidate the role of an additional epigenetic mechanism in gene expression alterations in NASH-related hepatocarcinogenesis, the status of H3K4me3, a well-established transcription activating modification (Santos-Rosa et al., 2002), was investigated in the promoter region of the 35 genes shown in Figure 1B. In contrast to gene-specific cytosine DNA methylation changes, the most prominent histone H3K4me3 alterations, in terms of significance, fold-change, and number of genes, were found in the NAFL, with the extent of histone H3K4me3 gradually diminishing during the progression of hepatocarcinogenesis and the development of HCC (Figure 2A and Supplementary Table 3). This was evidenced by the fact that the number of genes, especially those that exhibited a ≥2.0-fold change in gene-specific histone H3K4me3, decreased from 27 in NAFL (6 weeks) to nine in NASH-fibrotic (12 weeks) livers and five in full-fledged HCC (20 weeks). Likewise, the number of genes, the expression of which correlated with the level of gene-specific histone H3K4me3 enrichment, decreased with the progression of liver carcinogenesis (Figure 2B).

### Alterations of the *TUBB2B* Gene in Human Liver HepG2 and HepaRG Cells and in Human HCC

**FIGURE 3:**
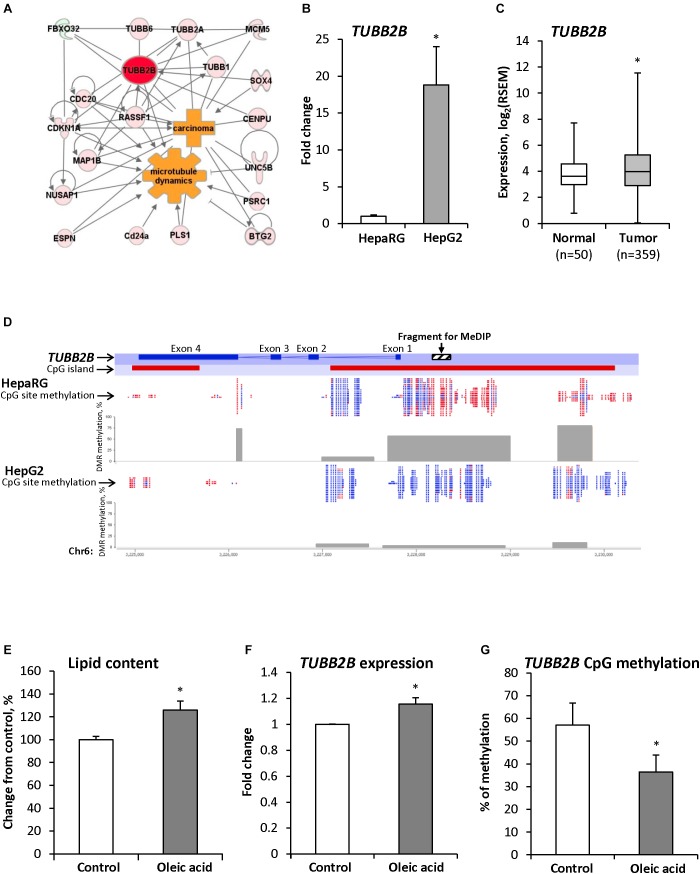
*TUBB2B* gene expression and methylation aberrations during hepatocarcinogenesis. **(A)** Molecular network of differentially expressed genes associated with *Tubb2b* and microtubule dynamics in HCC of mice subjected to STAM hepatocarcinogenesis. Red color indicates up-regulated genes and green color indicates down-regulated genes. **(B)** Expression of the *TUBB2B* gene in non-tumorigenic liver HepaRG cells and human HepG2 hepatocarcinoma cells. The *TUBB2B* gene expression is presented as an average fold change in HepG2 cells relative to that in the fully differentiated HepaRG cells, which was assigned value 1. **(C)** Expression of the *TUBB2B* gene in human HCC samples. **(D)** RRBS analysis of DNA methylation in the *TUBB2B* gene promoter region in HepaRG and HepG2 cells. Red color indicates methylated CpG sites and blue color indicates unmethylated CpG sites. Gray boxes indicate percent of methylated CpG sites from total number of CpG sites in the differentially methylated regions (DMR). **(E)** The level of triglycerides in the fully differentiated human hepatic HepaRG cells after culturing cells the presence of 250 μM oleic acid for 14 days. **(F,G)** Expression and promoter DNA methylation of the *TUBB2B* gene in fully differentiated HepaRG cells after culturing cells in the presence of 250 μM oleic acid for 14 days. The *TUBB2B* gene expression is presented as an average fold change in the HepaRG cells treated with oleic acid relative to the gene expression in non-treated HepaRG cells, which was assigned value 1. Values are mean ± SD, *n* = 3. ^∗^Denotes statistically a significant (*P* < 0.05) difference of gene expression and promoter DNA methylation.

To investigate the role of the *Tubb2b* gene over-expression in the pathogenesis of NASH and NASH-associated liver carcinogenesis, we performed a gene network interaction analysis and identified several co-regulated genes involved in microtubule dynamics and cytoskeleton organization (Figure 3A) that were up-regulated in NASH-derived HCC in STAM mice at 20 weeks (Supplementary Table 4).

Over-expression of the *TUBB2B* gene was also found in the human HCC HepG2 cell line as compared to non-tumorigenic HepaRG cells (Figure 3B) and in human HCC tissue samples (Figure 3C). In addition to the over-expression of *TUBB2B*, several other members of the tubulin family of genes were up-regulated in human HCC (Supplementary Figure 2). Figure 3D shows that over-expression of the *TUBB2B* gene in HepG2 hepatocarcinoma cells was accompanied by the promoter region demethylation as compared to that in HepaRG cells.

To confirm our *in vivo* findings of early up-regulation and demethylation of *Tubb2b* during NAFLD-associated liver carcinogenesis, we cultured human liver HepaRG cells in the presence of 250 μM oleic acid in the differentiation media for 14 days. We found that accumulation of triglycerides in HepaRG cells (Figure 3E) was accompanied by increased expression of *TUBB2B* (Figure 3F) and decreased methylation (by 21%) of the *TUBB2B* promoter region (Figure 3G). These findings provided independent evidence of the involvement of promoter DNA hypomethylation-associated over-expression of *TUBB2B* in the pathogenesis of NAFLD.

### Gene-Specific Methylation of Uniquely Differentially Expressed Genes in HCC

**FIGURE 4:**
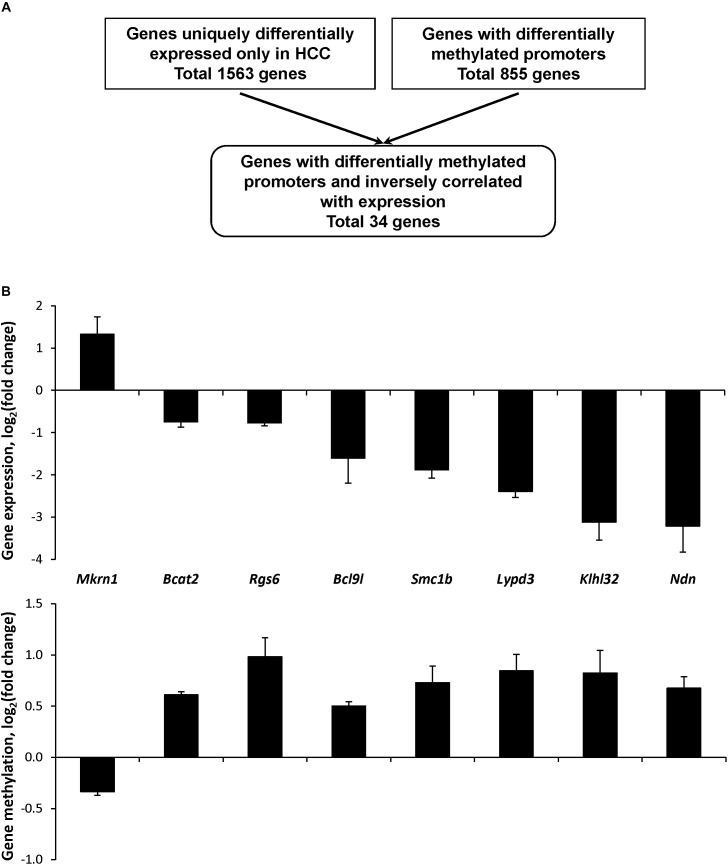
Expression and promoter DNA methylation of uniquely differentially expressed CpG island-containing genes in HCC in STAM mice at 20 weeks. **(A)** Algorithm for the selection of genes in HCC with differentially methylated promoters and inversely correlated with gene expression. **(B)** Gene expression and promoter DNA methylation of the selected genes differentially expressed in HCC. The results of gene expression and DNA methylation are presented as an average log_2_ fold change in the livers of mice with full-fledged HCC relative to respective values of age-matching control mice, which were assigned a value 1.

Considering the minimal association between gene expression and epigenetic alterations in the genes expressed in common during the hepatocarcinogenic process, the status of cytosine DNA methylation was investigated in genes differentially expressed only in HCC tissue samples. The results of high-throughput whole genome microarray gene expression and MeDIP microarray analyses demonstrated extensive gene expression and cytosine DNA methylation changes in the HCC samples. A total of 1563 genes was differentially expressed and 855 promoter CpG island-containing genes were differentially methylated; however, a combined analysis of differentially expressed and differentially methylated genes revealed a limited overlap in the number of differentially expressed and differentially methylated genes (Figure 4A). Specifically, only 34 genes exhibited an inverse correlation between expression and methylation (Supplementary Figure 3), among which only eight genes had a methylation level in HCC-tissues greater than 20% (Supplementary Table 5). Of these eight genes, seven were hypermethylated and down-regulated (Figure 4B).

To investigate the role of these genes uniquely expressed in HCC during the process of liver carcinogenesis, their expression and gene-specific methylation were investigated in NAFL and NASH-fibrotic tissues. No significant changes in the expression and cytosine methylation were found at the pre-HCC stages of liver carcinogenesis (Supplementary Table 5).

### Expression of Chromatin Modifying Genes in NASH-Related Hepatocarcinogenesis

**FIGURE 5:**
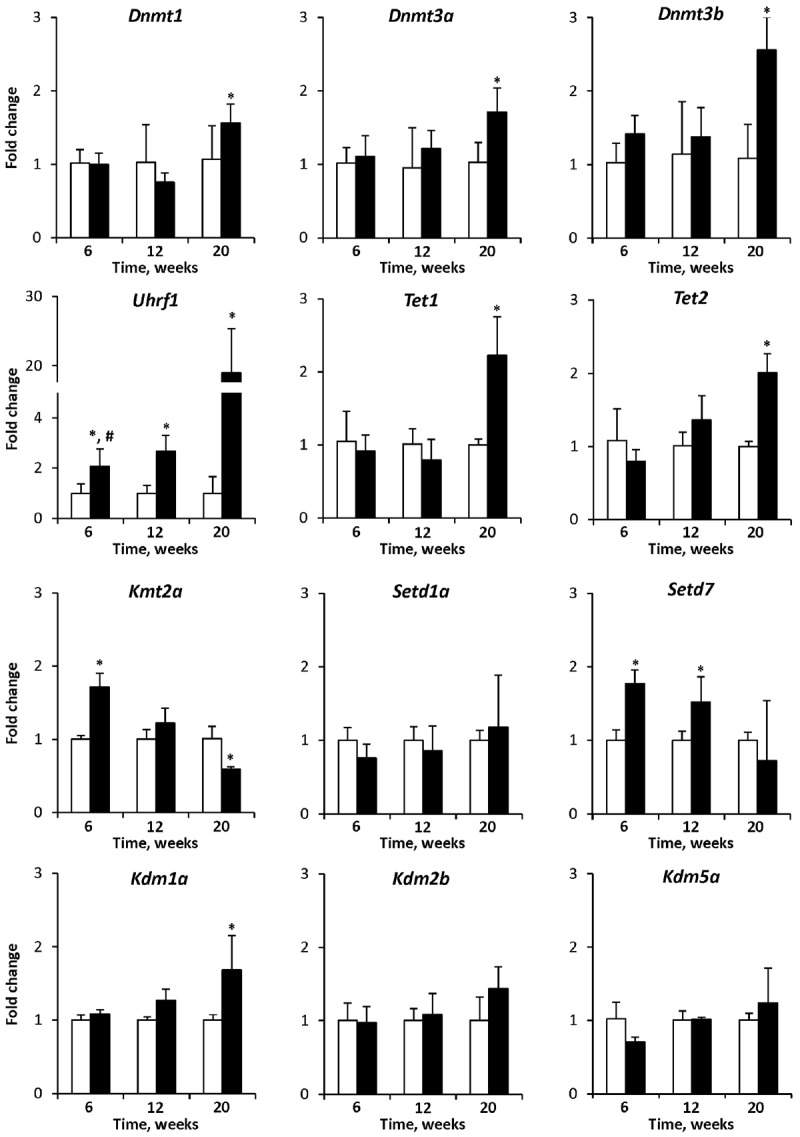
The expression of chromatin modifying genes in the livers of control mice and STAM mice subjected to NASH-derived hepatocarcinogenesis. The results are presented as an average fold change in the expression of each gene in the livers of STAM mice at 6, 12, and 20 weeks relative to that in control age-matching mice. The data are presented as the mean ± SD; *n* = 4. ^∗^Denotes a significant (*P* < 0.05) difference from the control age-matching group; ^#^denotes significant (*P* < 0.05) trend.

To investigate the mechanism of the observed cytosine DNA methylation and histone H3K4me3 alterations in the livers of STAM mice, the expression of chromatin modifying genes was investigated. The expression of the DNA methyltransferase genes *Dnmt1*, *Dnmt3a*, and *Dnmt3b* and the DNA demethylase genes *Tet1* and *Tet2* was significantly up-regulated in HCC samples only, while the expression of *Uhrf1* gradually increased throughout liver carcinogenesis (Figure 5). In contrast, the expression of histone H3K4 methylase genes, *Kmt2a*, and *Setd7*, gradually decreased during carcinogenesis, whereas the expression of the histone H3K4 demethylase gene *Kdm1a* increased, reflecting the dynamics of histone H3K4me3 alterations (Figure 5).

## Discussion

Profound alterations have been shown in HCC in the pattern of cytosine DNA methylation for multiple cancer-related genes, a well-recognized common mechanism of gene transcription regulation (Zhang et al., 2016; The Cancer Genome Atlas Research Network, 2017). This has led to the suggestion that aberrant cytosine DNA methylation may contribute to the hepatocarcinogenic process. Nonetheless, most human studies have provided a static snapshot of gene expression and gene-specific epigenetic alterations in HCC that does not necessarily clarify the relative contribution of these aberrations in the hepatocarcinogenic process.

In this study, using the STAM mouse model of NASH-derived liver carcinogenesis that depicts the sequential development of clinical and pathomorphological characteristics of NASH in diabetic patients, we demonstrated that NASH-related liver carcinogenesis is characterized by progressive accumulation of gene expression and gene-specific DNA methylation changes, with the greatest magnitude being found in full-fledged HCC; however, it is highly unlikely that all transcriptomic and DNA methylation aberrations found in full-fledged HCC may have significance in the development of HCC and its progression. To uncover alterations that may drive NASH-related liver carcinogenesis, we focused our investigation on 60 differentially expressed genes exhibiting the same trend of the expression changes at each stage of NASH-associated liver carcinogenesis. The results of our study showed that 35 out of 60 differentially expressed genes can be epigenetically regulated, as evidenced by the presence of CGIs in the promoter regions. Analysis of the promoter methylation status of these genes revealed that only the *Tubb2b* gene exhibited simultaneous methylation and gene expression changes during the carcinogenic process. This indicates that the gene expression changes in most of the differentially expressed genes are independent of promoter cytosine methylation and preceded the appearance and accumulation of gene-specific DNA methylation alterations in this model of liver carcinogenesis. This was further evidenced by a greater occurrence of gene promoter DNA methylation changes in full-fledged HCC compared to NAFL and NASH-fibrotic stages.

This finding is supported by growing evidence of the independence of gene expression changes from the gene promoter DNA methylation, especially at early stages of disease development or intervention. For instance, McKay et al. (2016) reported that feeding female C57BL/6J mice a low-folate (400 μg folic acid/kg) diet resulted in altered expression of 989 genes in male fetal liver, among which only 16 genes exhibited DNA methylation changes. Furthermore, several studies have demonstrated the existence of DNA methylation-independent changes in the expression of epigenetically regulated genes (Diesel et al., 2011; Espada et al., 2011; Navasa et al., 2015). Additionally, this finding is in good agreement with the suggestion that not all epigenetic alterations are equally important for the carcinogenic process; some may be drivers and trigger other molecular processes leading to neoplastic cell transformation, whereas others may be passenger events accompanying the transformation process and be a feature of transformed phenotype. In this respect, our finding of concurrent over-expression and promoter hypomethylation of the *Tubb2b* gene in this model of liver carcinogenesis is of special interest.

The *Tubb2* gene belongs to a gene family encoding several microtubule cytoskeleton α- and β-tubulin protein isotypes. Tubulins are essential for every eukaryotic cell, controlling cell shape, division, motility, and differentiation (Gadadhar et al., 2017). The fundamental mechanism that underlies a proper microtubule organization is the regulation of the levels of α- and β-tubulin isotypes. In normal cells, the composition of tubulin isotypes is tightly controlled and maintained, while in a broad range of cancer cells, including HCC cells, this composition is perturbed, and the expression of several tubulin isotypes is up-regulated. For example, Sun et al. (2007) demonstrated an over-expression of the *Tubb2*, *Tubb3*, and *Tubb6* genes and other cytoskeletal genes in HBV-related HCC in *Hbx* transgenic mice. Up-regulation of TUBA6, TUBA8, and TUBB3 has been reported in human liver cancer (Kuramitsu et al., 2011; Zen et al., 2014; Rein-Fischboeck et al., 2017). An increased expression of tubulin isotypes has been found not only in full-fledged HCC, but also in preneoplastic livers (Sun et al., 2007; Rein-Fischboeck et al., 2017). These findings are in good correspondence with the results of our study that showed a stage-dependent *Tubb2b* over-expression during NASH-associated liver carcinogenesis and the up-regulation of the *TUBB2B* gene in HepaRG cells subjected to the oleic acid-induced model of NAFL.

It is well-established that the regulation of the tubulin isotype composition, the “tubulin code,” is mediated by (i) expression of different α- and β-tubulin isotypes and (ii) post-transcriptional modifications of tubulins (Gadadhar et al., 2017). While the mechanisms of post-translational tubulin modifications are well-investigated, mechanism of transcriptional regulation of tubulin gene expression is less well studied. In view of this, the result of our study showing the involvement of epigenetic mechanisms in the regulation of *Tubb2b* expression, which was evidenced by simultaneous and progressive *Tubb2b* over-expression and gene promoter DNA hypomethylation during hepatocarcinogenesis and in HepaRG cells subjected to the oleic acid-induced model of NAFL, are of special interest. This finding is in good agreement with growing evidence on the role of DNA demethylation, including genome-wide (Yamada et al., 2005), global enhancer (Xiong et al., 2019), microRNA (Nojima et al., 2016), and gene-specific (Li et al., 2019) hypomethylation in the pathogenesis of HCC.

In summary, this report shows that the development of NASH-derived HCC is characterized by progressive accumulation of DNA methylation and gene expression alterations, with the greatest level of these abnormalities being found in full-fledged HCC. The results of the study illustrate that not all DNA methylation changes have an equal importance in the carcinogenic process. This was evidenced by the fact that majority of gene-specific DNA methylation changes occurred at the later stages of hepatocarcinogenesis, especially in full-fledged HCC, and were preceded by gene expression changes. In this respect, the observed concurrent and progressive *Tubb2b* expression and promoter methylation changes during the development of NASH-associated liver carcinogenesis are of great importance and indicate that unique *Tubb2b* gene-expression alterations mediated by aberrant DNA methylation may contribute to the development of HCC and may be used as the disease-specific indicator. Nevertheless, this study, as well as other studies using only male mouse models of NASH-associated liver carcinogenesis only (Asgharpour et al., 2016; Tsuchida et al., 2018), does not provide the answer whether similar alterations exist in female mice. Therefore, future studies are needed to address this question. The results of these studies may identify early sex-independent diagnostic biomarkers of NASH that may be useful for monitoring disease stratification.

## Ethics Statement

All experimental procedures were performed according to the Japanese Pharmacological Society Guidelines and experimental protocols were approved by the SMC Laboratories, Inc., Research Animal Care and Use Committee.

## Author Contributions

AC, FB, and IP designed the study. KD, VT, and AC performed the experiments. All authors participated in analyzing and interpretation of the data, discussion of the results, and writing of the manuscript.

## Disclaimer

The views expressed in this manuscript do not necessarily represent those of the U.S. Food and Drug Administration.

## Conflict of Interest Statement

The authors declare that the research was conducted in the absence of any commercial or financial relationships that could be construed as a potential conflict of interest.

## Footnotes

1 http://cancergenome.nih.gov

## Abbreviations

CGIs: CpG islands
ChIP: chromatin immunoprecipitation
FDR: false discovery rate
GEO: Gene Expression Omnibus
H3K4me3: histone H3 lysine 4 trimethylation
HBV: hepatitis B virus
HCC: hepatocellular carcinoma
HCV: hepatitis C virus
MeDIP: methylated DNA immunoprecipitation
NAFLD: non-alcoholic fatty liver disease
NASH: non-alcoholic steatohepatitis
PBS: phosphate-buffered saline
qPCR: quantitative PCR
RRBS: Reduced Representation Bisulfite Sequencing
RSEM: RNA-Seq by Expectation Maximization
STAM: Stelic Animal Model
TCGA: The Cancer Genome Atlas.

## References

[B1] Asgharpour A. Cazanave S. C. Pacana T. Seneshaw M. Vincent R. Banini B. A. (2016). A diet-induced animal model of non-alcoholic fatty liver disease and hepatocellular cancer. *J. Hepatol.* 65 579–588. 10.1016/j.jhep.2016.05.005 27261415 PMC5012902

[B2] Baylin S. Bestor T. H. (2002). Altered methylation patterns in cancer cell genomes: cause or consequence? *Cancer Cell* 1 299–305. 10.1016/S1535-6108(02)00061-2 12086841

[B3] Benjamini Y. Hochberg Y. (1995). Controlling the false discovery rate: a practical and powerful approach to multiple testing. *J. R. Stat. Soc. Series B Stat. Methodol.* 57 289–300.

[B4] Bertuccio P. Turati F. Carioli G. Rodriguez T. La Vecchia C. Malvezzi M. (2017). Global trends and predictions in hepatocellular carcinoma mortality. *J. Hepatol.* 67 302–309. 10.1016/j.jhep.2017.03.011 28336466

[B5] Bestor T. H. Edwards J. R. Boulard M. (2015). Notes on the role of dynamic DNA methylation in mammalian development. *Proc. Natl. Acad. Sci. U.S.A.* 112 6796–6799. 10.1073/pnas.1415301111 25368180 PMC4460495

[B6] Choo S. P. Tan W. L. Goh B. K. P. Tai W. M. Zhu A. X. (2016). Comparison of hepatocellular carcinoma in Eastern versus Western populations. *Cancer* 122 3430–3446. 10.1002/cncr.30237 27622302

[B7] de Conti A. Dreval K. Tryndyak V. Orisakwe O. E. Ross S. A. Beland F. A. (2017). Inhibition of the cell death pathway in nonalcoholic steatohepatitis (NASH)-related hepatocarcinogenesis is associated with histone H4 lysine 16 deacetylation. *Mol. Cancer. Res.* 15 1163–1172. 10.1158/1541-7786.MCR-17-0109 28512251

[B8] Diesel B. Ripoche N. Risch R. T. Tierling S. Walter J. Kiemer A. K. (2011). Inflammation-induced up-regulation of TLR2 expression in human endothelial cells is independent of differential methylation in the TLR2 promoter CpG island. *Innate Immun.* 18 112–123. 10.1177/1753425910394888 21768203

[B9] Edwards J. R. Yarychkivska O. Boulard M. Bestor T. H. (2017). DNA methylation and DNA methyltransferases. *Epigenetics Chromatin* 10:23. 10.1186/s13072-017-0130-8 28503201 PMC5422929

[B10] Ehrlich M. Lacey M. (2013). DNA methylation and differentiation: silencing, upregulation and modulation of gene expression. *Epigenomics* 5 553–568. 10.2217/epi.13.43 24059801 PMC3864898

[B11] Espada J. Peinado H. Lopez-Serra L. Setién F. Lopez-Serra P. Portela A. (2011). Regulation of SNAIL1 and E-cadherin function by DNMT1 in a DNA-methylation-independent context. *Nucleic Acids Res.* 39 9194–9205. 10.1093/nar/gkr658 21846773 PMC3241660

[B12] Fujii M. Shibazaki Y. Wakamatsu K. Honda Y. Kawauchi Y. Suzuki K. (2013). A murine model for non-alcoholic steatohepatitis showing evidence of association between diabetes and hepatocellular carcinoma. *Med. Mol. Morphol.* 46 141–152. 10.1007/s00795-013-0016-1 23430399

[B13] Gadadhar S. Bodakuntla S. Natarajan K. Janke C. (2017). The tubulin code at a glance. *J. Cell. Sci.* 130 1347–1353. 10.1242/jcs.199471 28325758

[B14] Jones P. A. (2012). Functions of DNA methylation: islands, start sites, gene bodies and beyond. *Nat. Rev. Genet.* 13 484–492. 10.1038/nrg3230 22641018

[B15] Kim D. Li A. A. Perumpail B. J. Gadiparthi C. Kim W. Cholankeril G. (2019). Changing trends in etiology-based and ethnicity-based annual mortality rates of cirrhosis and hepatocellular carcinoma in the United States. *Hepatology* 69 1064–1074. 10.1002/hep.30161 30014489 PMC6709988

[B16] Kuramitsu Y. Takashima M. Yokoyama Y. Iizuka N. Tamesa T. Akada J. K. (2011). Up-regulation of 42 kDa tubulin alpha-6 chain fragment in well-differentiated hepatocellular carcinoma tissues from patients infected with hepatitis C virus. *Anticancer Res.* 31 3331–3336. 21965743

[B17] Li B. Dewey C. N. (2011). RSEM: accurate transcript quantification from RNA-Seq data with or without a reference genome. *BMC Bioinformatics* 12:323. 10.1186/1471-2105-12-323 21816040 PMC3163565

[B18] Li Z. Li Z. Wang L. Long C. Zheng Z. Zhuang X. (2019). ZCCHC13-mediated induction of human liver cancer is associated with the modulation of DNA methylation and the AKT/ERK signaling pathway. *J. Transl. Med.* 17:108. 10.1186/s12967-019-1852-0 30940166 PMC6444591

[B19] Llovet J. M. Zucman-Rossi J. Pikarsky E. Sangro B. Schwartz M. Sherman M. (2016). Hepatocellular carcinoma. *Nat. Rev. Dis. Primers* 2:16018. 10.1038/nrdp.2016.18 27158749

[B20] Long M. D. Smiraglia D. J. Campbell M. J. (2017). The genomic impact of DNA CpG methylation on gene expression; relationships in prostate cancer. *Biomolecules* 7:15. 10.3390/biom7010015 28216563 PMC5372727

[B21] Marengo A. Rosso C. Bugianesi E. (2016). Liver cancer: connections with obesity, fatty liver, and cirrhosis. *Annu. Rev. Med.* 67 103–117. 10.1146/annurev-med-090514-013832 26473416

[B22] McKay J. A. Adriaens M. Evelo C. T. Ford D. Mathers J. C. (2016). Gene promoter DNA methylation patterns have limited role in orchestrating transcriptional changes in the fetal liver in response to maternal folate depletion during pregnancy. *Mol. Nutr. Food Res.* 60 2031–2042. 10.1002/mnfr.201600079 27133805 PMC5031189

[B23] Murphy S. K. Yang H. Moylan C. A. Pang H. Dellinger A. Abdelmalek M. F. (2013). Relationship between methylome and transcriptome in patients with nonalcoholic fatty liver disease. *Gastroenterology* 145 1076–1087. 10.1053/j.gastro.2013.07.047 23916847 PMC3805742

[B24] Navasa N. Martin-Ruiz I. Atondo E. Sutherland J. D. Pascual-Itoiz M. A. Carreras-González A. (2015). Ikaros mediates the DNA methylation-independent silencing of MCJ/DNAJC15 gene expression in macrophages. *Sci. Rep.* 5:14692. 10.1038/srep14692 26419808 PMC4588509

[B25] Nojima M. Matsui T. Tamori A. Kubo S. Shirabe K. Kimura K. (2016). Global, cancer-specific microRNA cluster hypomethylation was functionally associated with the development of non-B non-C hepatocellular carcinoma. *Mol. Cancer* 15:31. 10.1186/s12943-016-0514-6 27137948 PMC4852433

[B26] Pogribny I. P. Rusyn I. (2014). Role of epigenetic aberrations in the development and progression of human hepatocellular carcinoma. *Cancer Lett.* 342 223–230. 10.1016/j.canlet.2012.01.038 22306342 PMC3971756

[B27] Rein-Fischboeck L. Pohl R. Haberl E. M. Zimny S. Neumann M. Eisinger K. (2017). Tubulin alpha 8 is expressed in hepatic stellate cells and is induced in transformed hepatocytes. *Mol. Cell. Biochem.* 428 161–170. 10.1007/s11010-016-2926-4 28063004

[B28] Rogue A. Anthérieu S. Vluggens A. Umbdenstock T. Claude N. de la Moureyre-Spire C. (2014). PPAR agonists reduce steatosis in oleic acid-overloaded HepaRG cells. *Toxicol. Appl. Pharmacol.* 276 73–81. 10.1016/j.taap.2014.02.001 24534255

[B29] Santos-Rosa H. Schneider R. Bannister A. J. Sherriff J. Bernstein B. E. Emre N. C. T. (2002). Active genes are tri-methylated at K4 of histone H3. *Nature* 419 407–411. 10.1038/nature01080 12353038

[B30] Schmittgen T. D. Livak K. J. (2008). Analyzing real-time PCR data by the comparative C_T_ method. *Nat. Protoc.* 3 1101–1108. 10.1038/nprot.2008.73 18546601

[B31] Sun Q. Wang Y. Zhang Y. Liu F. Cheng X. Hou N. (2007). Expression profiling reveals dysregulation of cellular cytoskeletal genes in HBx-induced hepatocarcinogenesis. *Cancer Biol. Ther.* 6 668–674. 10.4161/cbt.6.5.3955 17873514

[B32] Takai D. Jones P. A. (2002). Comprehensive analysis of CpG islands in human chromosomes 21 and 22. *Proc. Natl. Acad. Sci. U.S.A.* 99 3740–3745. 10.1073/pnas.052410099 11891299 PMC122594

[B33] Takakura K. Koido S. Fujii M. Hashiguchi T. Shibazaki Y. Yoneyama H. (2014). Characterization of non-alcoholic steatohepatitis-derived hepatocellular carcinoma as a human stratification model in mice. *Anticancer Res.* 34 4849–4855. 25202066

[B34] The Cancer Genome Atlas Research Network. (2017). Comprehensive and integrative genomic characterization of hepatocellular carcinoma. *Cell* 169 1327–1341. 10.1016/j.cell.2017.05.046 28622513 PMC5680778

[B35] Tryndyak V. de Conti A. Doerge D. R. Olson G. R. Beland F. A. Pogribny I. P. (2017). Furan-induced transcriptomic and gene-specific DNA methylation changes in the livers of Fischer 344 rats in a 2-year carcinogenicity study. *Arch. Toxicol.* 91 1233–1243. 10.1007/s00204-016-1786-8 27387713

[B36] Tryndyak V. P. Han T. Fuscoe J. C. Ross S. A. Beland F. A. Pogribny I. P. (2016). Status of hepatic DNA methylome predetermines and modulates the severity of non-alcoholic fatty liver injury in mice. *BMC Genomics* 17:298. 10.1186/s12864-016-2617-2 27103143 PMC4840954

[B37] Tsuchida T. Lee Y. A. Fujiwara N. Ybanez M. Allen B. Martins S. (2018). A simple diet- and chemical-induced murine NASH model with rapid progression of steatohepatitis, fibrosis and liver cancer. *J. Hepatol.* 69 385–395. 10.1016/j.jhep.2018.03.011 29572095 PMC6054570

[B38] Villanueva A. Portela A. Sayols S. Battiston C. Hoshida Y. Méndez-González J. (2015). DNA methylation-based prognosis and epidrivers in hepatocellular carcinoma. *Hepatology* 61 1945–1956. 10.1002/hep.27732 25645722 PMC12337117

[B39] Xiong L. Wu F. Wu Q. Xu L. Cheung O. K. Kang W. (2019). Aberrant enhancer hypomethylation contributes to hepatic carcinogenesis through global transcriptional reprogramming. *Nat. Commun.* 10:335. 10.1038/s41467-018-08245-z 30659195 PMC6338783

[B40] Yamada N. Yasui K. Dohi O. Gen Y. Tomie A. Kitaichi T. (2016). Genome-wide DNA methylation analysis in hepatocellular carcinoma. *Oncol. Rep.* 35 2228–2236. 10.3892/or.2016.4619 26883180

[B41] Yamada Y. Jackson-Grusby L. Linhart H. Meissner A. Eden A. Lin H. (2005). Opposing effects of DNA hypomethylation on intestinal and liver carcinogenesis. *Proc. Natl. Acad. Sci. U.S.A.* 102 13580–13585. 10.1073/pnas.0506612102 16174748 PMC1224663

[B42] Younes R. Bugianesi E. (2018). Should we undertake surveillance for HCC in patients with NAFLD? *J. Hepatol.* 68 326–334. 10.1016/j.jhep.2017.10.006 29122695

[B43] Younossi Z. M. Otgonsuren M. Henry L. Venkatesan C. Mishra A. Erario M. (2015). Association of nonalcoholic fatty liver disease (NAFLD) with hepatocellular carcinoma (HCC) in the United States from 2004 to 2009. *Hepatology* 62 1723–1730. 10.1002/hep.28123 26274335

[B44] Zen Y. Britton D. Mitra V. Pike I. Sarker D. Itoh T. (2014). Tubulin β-III: a novel immunohistochemical marker for intrahepatic peripheral cholangiocarcinoma. *Histopathology* 65 784–792. 10.1111/his.12497 25039376

[B45] Zhang C. Li J. Huang T. Duan S. Dai D. Jiang D. (2016). Meta-analysis of DNA methylation biomarkers in hepatocellular carcinoma. *Oncotarget* 7 81255–81267. 10.18632/oncotarget.13221 27835605 PMC5348390

[B46] Zucman-Rossi J. Villanueva A. Nault J.-C. Llovet J. M. (2015). Genetic landscape and biomarkers of hepatocellular carcinoma. *Gastroenterology* 149 1226–1239. 10.1053/j.gastro.2015.05.061 26099527

