# Supplementary figures and images for: Gene Expression and DNA Methylation Alterations During Non-alcoholic Steatohepatitis-Associated Liver Carcinogenesis
Source: Front Genet. 2019 May 29;10:486. doi: 10.3389/fgene.2019.00486 (PMC6549534; doi:10.3389/fgene.2019.00486)

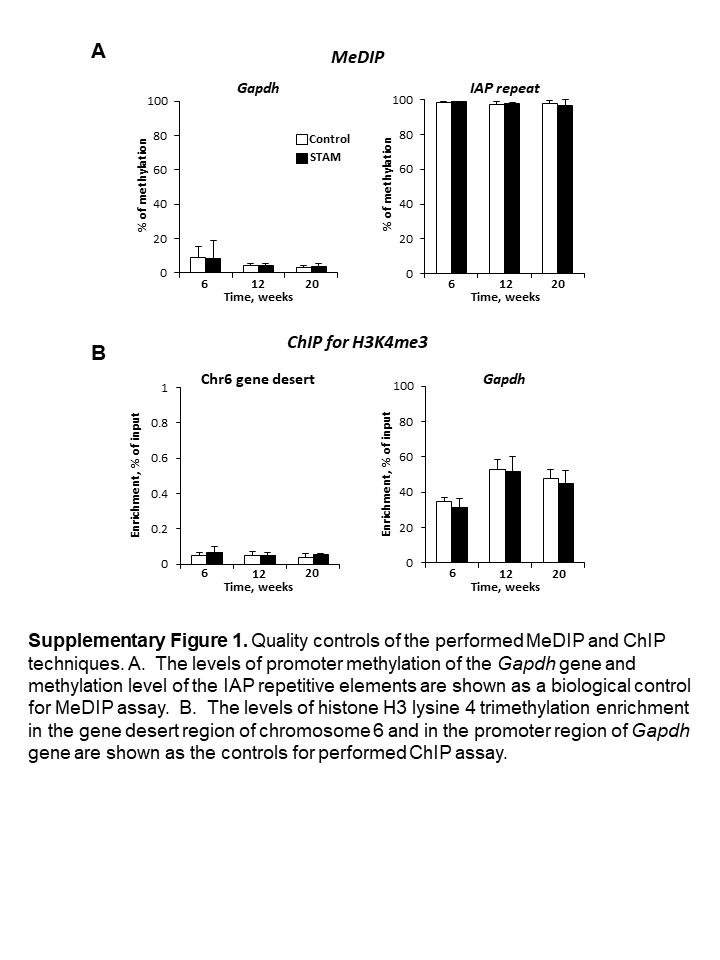

Supplement: Supplementary file 1 [file Image_1.TIF]

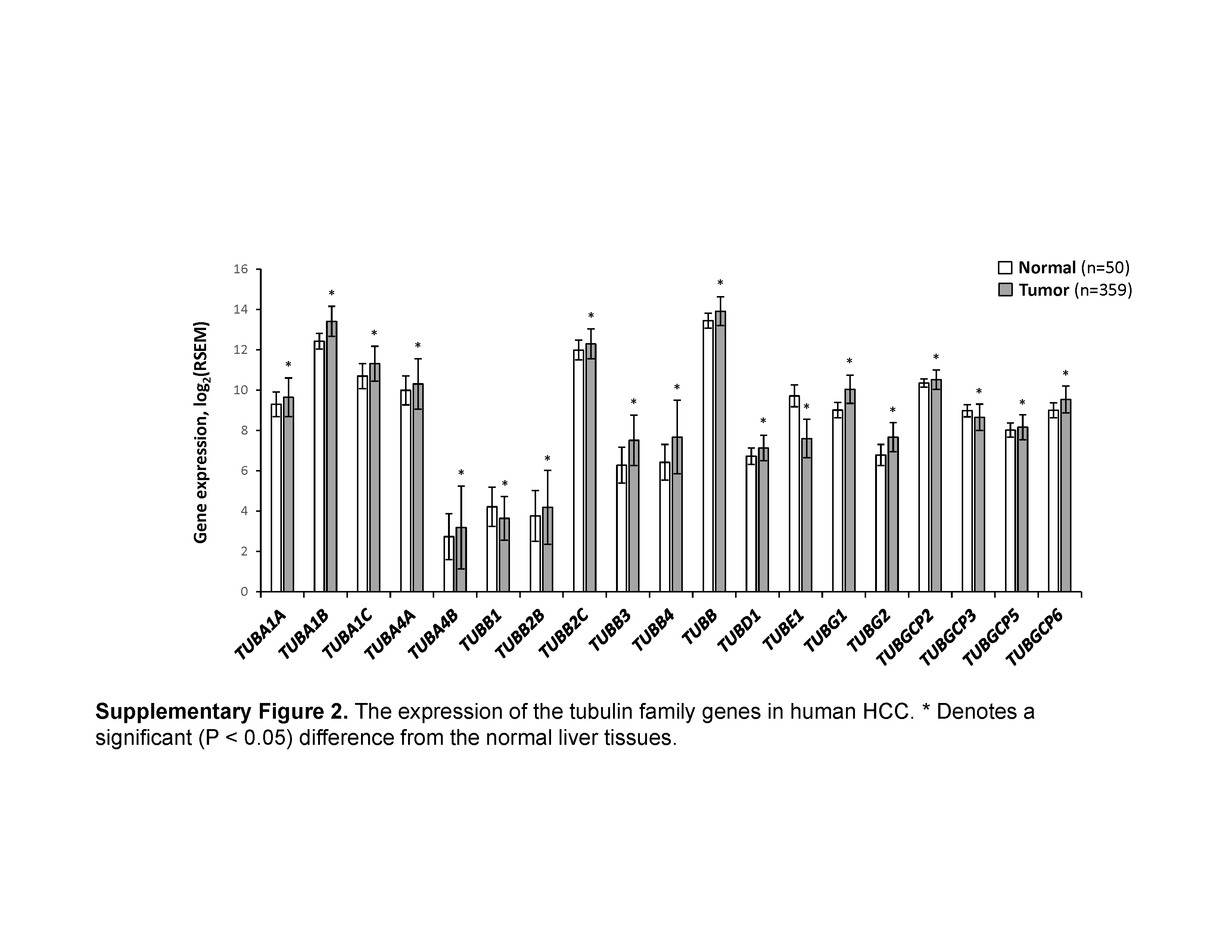

Supplement: Supplementary file 2 [file Image_2.TIF]
