# Supplementary material for: Gene Expression and DNA Methylation Alterations During Non-alcoholic Steatohepatitis-Associated Liver Carcinogenesis
Source: Front Genet. 2019 May 29;10:486. doi: 10.3389/fgene.2019.00486 (PMC6549534; doi:10.3389/fgene.2019.00486)
Supplement: Supplementary file 4 [file Table_1.DOC]

**Supplementary Table 1.** Primers used in the study.

| **TaqMan primers used for qRT-PCR** | | |
| --- | --- | --- |
| **Target gene** | | **Assay ID** |
| ***Dnmt1*** | | Mm01151063_m1 |
| ***Dnmt3a*** | | Mm00432881_m1 |
| ***Dnmt3b*** | | Mm01240113_m1 |
| ***Kdm1a*** | | Mm01181029_m1 |
| ***Kdm2b*** | | Mm01194587_m1 |
| ***Kdm5a*** | | Mm00524457_m1 |
| ***Kmt2a*** | | Mm01179235_m1 |
| ***Setd1a*** | | Mm00626141_m1 |
| ***Setd7*** | | Mm00499823_m1 |
| ***Tbp*** | | Mm01277042_m1 |
| ***Tet1*** | | Mm01169087_m1 |
| ***Tet2*** | | Mm00524395_m1 |
| ***Uhrf1*** | | Mm00477872_m1 |
| **Custom designed primers used for qPCR reactions in MeDIP and ChIP assays** | | |
| **Name** | **Primer** | **Sequence, 5’ to 3’** |
| ***Mouse primers*** |  |  |
| ***Aldh3a2*** | FW | TGCCAAAAATTGCACCTACA |
|  | RV | AGCTGCTGCACGTGACTATG |
| ***Bcat2*** | FW | AGTGTGGCTGTGGTAGAGCA |
|  | RV | CCAGGCTCTGCTGTGAAGAT |
| ***Bcl9l*** | FW | TCTCTACACTCGGGCTTTGC |
|  | RV | TAACGCTGGCTCAGATTCCT |
| ***Bmp8b*** | FW | CCTGGCAGAGAATAGCTTGG |
|  | RV | CCTGTCTGCTAGGTGCCTTC |
| ***Btg2*** | FW | ATCCCCATTGCTGAAAACAC |
|  | RV | GACACTGACAGAGCCGTTCA |
| ***Cadm4*** | FW | CCTGCCCAAAGAAGTCCATA |
|  | RV | AGGAGTGTGATGATGCCACA |
| ***Cbr3*** | FW | CCCTGACCACTGCTTAGCTC |
|  | RV | CTGAGGAGGAGCCGTCTGT |
| ***Ccnb2*** | FW | GCCTTCCAGTCTAGCCAATG |
|  | RV | GGTCCGCGATTCAAATACC |
| ***Cd24a*** | FW | GGGTGGAAATGGCTTGAATA |
|  | RV | TCGGTCCCAGTATTCTCCAC |
| ***Cdc20*** | FW | AGAGATGGGTTCGGGATTTC |
|  | RV | CGATTGGTCGCTACACTGAA |
| ***Cgref1*** | FW | CGCGGTGCCTATAGAGTTCA |
|  | RV | GGAGTCTGACCCACCTGCT |
| ***Cmtm6*** | FW | CGGAGTCCCAGGAAGTGAC |
|  | RV | CTGTAGACCGCTCCGTTCTC |
| ***Dll4*** | FW | GAAGCCCAGAAGTTGGAAGA |
|  | RV | AGTGGCTACAGTCCCAGCAC |
| ***Dnajb11*** | FW | ACATGGCGTAGCTTCCAGAC |
|  | RV | GTAGGAAACCCGAATGTCCA |
| ***Dusp10*** | FW | AGTCGGGCTCCTGAGAACAG |
|  | RV | CGTGCTCTTGTTCCCCTACT |
| ***Eid2*** | FW | AGACACGCGACCCCCTAC |
|  | RV | GCTGAGACCCGGGAAGAAC |
| ***Elovl7*** | FW | TCAGTGAAGGAGACGGGAGT |
|  | RV | GGGAGGCTTTTAGTGCAATG |
| ***Epdr1*** | FW | GAAAATCGAGAGAGGCCTGA |
|  | RV | GACTAGTCGCGTGAGCCTTC |
| ***Espn*** | FW | GTCCCCAGCAACCTAAAACA |
|  | RV | TGCGCACAAAACCTGAATAG |
| ***Fbxo31*** | FW | TCAGGTCTGCAGCAGGTTC |
|  | RV | GGCCAAGTGTCCGTACTAGG |
| ***Fbxo5*** | FW | TGGGCACCAAATTCAAAGAT |
|  | RV | GCGACGACAGGAAAAAGGT |
| ***Fryl*** | FW | AATCAGAGTCGGAGGGAGGT |
|  | RV | CAGGCTCCGTTCTCTTTGAC |
| ***Fst*** | FW | CCGCTCCTACGCAAATAAGA |
|  | RV | CATTTCCCACCTTCTGGAAA |
| ***Gdf10*** | FW | GCTCAGGATAGCCGTGGTC |
|  | RV | GAGGTATCGGGATTGGCTCT |
| ***Ggta1*** | FW | CTTCAGTGGGGAGGAGGTGT |
|  | RV | TGATCGAGTGACCAGGACAA |
| ***Gipc2*** | FW | TCCATCCTTTCGTTTTCCTG |
|  | RV | AGCCTGGCCTACAGAGTGAG |
| ***Hells*** | FW | GTTTTCCCGCGAAAGAGAAG |
|  | RV | GCGAGCTAAATCCTCACACC |
| ***Ints2*** | FW | GCCCTAAGCTGGCTTTTCTT |
|  | RV | TCCATTCATCCCAAGCTTCT |
| ***Kbtbd11*** | FW | GGATGTGGGCAACAGATTTT |
|  | RV | GCTCTTGGCTGTTTGACCTC |
| ***Klhl32*** | FW | CTTTCTCTTCCTGCCGACAC |
|  | RV | CTCTGGCGTGTGTGTGGAT |
| ***Lect1*** | FW | GGCCTAGGGAAGAGTCTGCT |
|  | RV | GCGAGGACTGGAGTAGGAAA |
| ***Lepr*** | FW | TCTCTTTCCCACTTGGCACT |
|  | RV | AGCTTTGAGGGCTGATTCTG |
| ***Lhx2*** | FW | CCTAGCTGTTCCTGGGTGAA |
|  | RV | ATCGCTAGCTGGGTTCTGG |
| ***Lmo2*** | FW | ACCGGGATGGAAGGTTAAGT |
|  | RV | CCTTCAAACGCCAAAGAGAG |
| ***Lypd3*** | FW | GACTACCCCTTCCCAACTCC |
|  | RV | GGTCTCAGTGAGGGCTGAAA |
| ***Mest*** | FW | GGTGACTTTGGCCCATTCTA |
|  | RV | GGCACACTAAATGGCTAGGG |
| ***Mier1*** | FW | GCTGCCCACCTGTTGTCC |
|  | RV | CCTCCCAACTGCCTCAAGT |
| ***Mkrn1*** | FW | CCTCACCTCTATCCCCACAG |
|  | RV | AGGTGACCTGTTTCGTCCAG |
| ***Mxd3*** | FW | GCTATCAACAGCCGACGTTT |
|  | RV | CGTATCTGCCCTTCATTGGT |
| ***Ndn*** | FW | CCTACCACCCTTCTGGCTTC |
|  | RV | GGAAGTGCGCTTTACTGAGC |
| ***Nfkb2*** | FW | AAGCCTGGAAGAGTGCAGAG |
|  | RV | ACCCAGCTCCAGTTCTGCTA |
| ***Nudt18*** | FW | ACCCTACAGGCAGGATGACA |
|  | RV | CTGGGTTTCAGCCTCACTTC |
| ***Nuf2*** | FW | GGAACTCCTCCCCCTAACTG |
|  | RV | CTCCAGATCAGCGGAATCAT |
| ***Nusap1*** | FW | GCCTCACTCACTGGAGAAGG |
|  | RV | GAGAGATGTGCCGTGTTTCA |
| ***Pdgfb*** | FW | AGCTCTGCGCTTTCTGATCT |
|  | RV | GCTAAAGGCGTGTTCCTCTG |
| ***Phlda3*** | FW | TCTGAGAAGCAGCGGGTATT |
|  | RV | TAGAGGAGCCAGGTCATTGC |
| ***Pim3*** | FW | CGAGACTCAAAACAGCCACA |
|  | RV | TTGGACCACGGAGATGTGTA |
| ***Plekhh1*** | FW | TGCTGAGGTCATTTCCTGTG |
|  | RV | GTCGGAGCCTGGAAGGTC |
| ***Pls1*** | FW | ACTAAAACCTGGCGAGTGGA |
|  | RV | CACTCACCCGCCTCCTTG |
| ***Ppm1h*** | FW | AGGTCACGATCTCGGAGCTA |
|  | RV | ATGTGATGATCAGGCTGCAC |
| ***Pqlc3*** | FW | TCTTGAGTGGAGCAGACTCG |
|  | RV | GGAGGCTCAAAGTCCAAAAA |
| ***Procr*** | FW | AGAGTCGGCAACATCGAAAG |
|  | RV | CTTGAGGAGGAGCGGAAAAG |
| ***Psrc1*** | FW | GCAAGGAAACCCAGTCTCAG |
|  | RV | CGAGTCAGACAAGGGGAGAA |
| ***Rbpms2*** | FW | CCCGCCAAACTTAATGTGAT |
|  | RV | GAGAGTGCGAGCTGGTGAG |
| ***Rgs6*** | FW | GGTGGAAGAGGAGGAGAAGG |
|  | RV | TCATGATAAGGCTGGGGAAC |
| ***Samd4*** | FW | TGCCAAGCCCATGATGTAAT |
|  | RV | CACTGCTGCCTCCAGTGTC |
| ***Sash1*** | FW | TCTGCTCCAGCTACCCTGTT |
|  | RV | CTGGGTACGAGCACCACTG |
| ***Slc25a37*** | FW | CCAGCTATTGGTGCTTCTGG |
|  | RV | GAGGGGGAGGAAAACTTCAC |
| ***Slc35f2*** | FW | ACTAGAGAGCGCAGCAGGAG |
|  | RV | GGAACCCCGAGAAGTGCT |
| ***Slc7a1*** | FW | GTCATTGGTGCCCTGGAA |
|  | RV | AATCCGAGCCGGTTTCAT |
| ***Smc1b*** | FW | TTGGCCTCCTGTTGTAGGTC |
|  | RV | GCTTTTCTTGCCGTGTTTTT |
| ***Smox*** | FW | GGAAGACCTCGTCTGCTTTG |
|  | RV | CACTCCCCTATCTCGCAAAA |
| ***Sqstm1*** | FW | TAGTAGGTCTGGGCGAGGAC |
|  | RV | CGAACCGCTGGATGTTAGAT |
| ***Tnfrsf23*** | FW | ACAGAAACCAATCCGCTGAC |
|  | RV | TGAAGGTAACCATGGCTGTG |
| ***Tubb2b*** | FW | GAGGCACTACTCTCGCCAAG |
|  | RV | TGAGCACTGGTAGGGAGCTT |
| ***Uap1l1*** | FW | GGTGCTCTTACCCACTGAGC |
|  | RV | GCCTTCCTAGTACCCCAAGG |
| ***Unc5b*** | FW | GCTGGAACTCCCCAACTACA |
|  | RV | TTTCGCTTTTGATTCGGTCT |
| ***Usp18*** | FW | GCTTGTCGTGTCCTGGTGTA |
|  | RV | CCAAAGGAAAGCGAAACTCA |
| ***Vasp*** | FW | TGCCTTCTGGAACGATCTCT |
|  | RV | GCCATCAAGAGAACCTCAGC |
| ***Gapdh*** | FW | GGACTGCCTGGTGTCCTTC |
|  | RV | CCGACCTTCACCATTTTGTC |
| ***IAP repeats*** | FW | AGCAGGTGAAGCCACTG |
|  | RV | CTTGCCACACTTAGAGC |
| ***Human primers*** |  |  |
| ***TUBB2B* (Fragment for MeDIP)** | FW | TGGGGGAACAGTGAAGAAAA |
|  | RV | ACATGCTCCAGTCCTCCATC |
