# Supplementary material for: Gene Expression and DNA Methylation Alterations During Non-alcoholic Steatohepatitis-Associated Liver Carcinogenesis
Source: Front Genet. 2019 May 29;10:486. doi: 10.3389/fgene.2019.00486 (PMC6549534; doi:10.3389/fgene.2019.00486)
Supplement: Supplementary file 6 [file Table_3.DOC]

**Supplementary Table 3.** Gene expression and gene-specific histone H3K4me3 of epigenetically regulated differentially expressed genes during NASH-associated liver carcinogenesis.

| **#** | **Gene name** | **Gene ID** | **Expression, fold change** | | | **H3K4me3, fold change** | | |
| --- | --- | --- | --- | --- | --- | --- | --- | --- |
|  |  |  | **NAFL, 6 weeks** | **NASH-fibrosis, 12 weeks** | **HCC, 20 weeks** | **NAFL, 6 weeks** | **NASH-fibrosis, 12 weeks** | **HCC, 20 weeks** |
| 1 | Tubb2b | NM_023716 | 9.1 | 24.7 | 401.8 | **7.3*** | 1.3 | 1.3 |
| 2 | Bmp8b | NM_007559 | 21.3 | 54.4 | 158.7 | **11.6** | **2.2** | **2.4** |
| 3 | Elovl7 | NM_029001 | 10.7 | 12.3 | 155.6 | **5.2** | 1.8 | 1.1 |
| 4 | Lepr | NM_001122899 | 10.6 | 29.6 | 52.0 | **3.6** | **3.1** | **2.5** |
| 5 | Gipc2 | NM_016867 | 6.9 | 49.0 | 51.6 | **8.2** | **2.0** | 1.9 |
| 6 | Psrc1 | NM_019976 | 20.9 | 28.3 | 47.7 | **3.7** | 1.6 | 1.2 |
| 7 | Pls1 | NM_001033210 | 12.5 | 14.7 | 33.9 | **3.0** | 1.8 | 1.3 |
| 8 | Epdr1 | NM_134065 | 17.4 | 16.1 | 22.9 | **3.6** | **2.7** | **2.2** |
| 9 | Nusap1 | NM_133851 | 2.4 | 4.9 | 19.1 | **2.0** | 1.3 | 1.2 |
| 10 | Cbr3 | NM_173047 | 11.0 | 10.9 | 15.9 | **2.3** | 1.5 | 1.4 |
| 11 | Phlda3 | NM_013750 | 6.1 | 9.3 | 14.6 | **6.6** | **2.3** | **2.3** |
| 12 | Cgref1 | NM_026770 | 7.0 | 14.8 | 14.6 | **3.2** | **2.6** | 1.6 |
| 13 | Cdc20 | NM_023223 | 2.2 | 2.1 | 13.0 | **3.1** | 1.5 | 1.3 |
| 14 | Plekhh1 | NM_181073 | 3.8 | 5.9 | 11.6 | 1.8 | 1.8 | 1.1 |
| 15 | Pqlc3 | NM_172574 | 5.4 | 5.3 | 9.7 | **2.7** | **2.2** | **2.0** |
| 16 | Cd24a | NM_009846 | 3.0 | 5.2 | 8.2 | **5.5** | **2.0** | 1.1 |
| 17 | Unc5b | NM_029770 | 2.9 | 3.0 | 6.8 | **2.0** | 1.2 | 0.8 |
| 18 | Btg2 | NM_007570 | 2.1 | 5.5 | 6.7 | **3.8** | 1.8 | 1.3 |
| 19 | Mest | NM_001252292 | 2.8 | 3.8 | 6.1 | **5.1** | 1.7 | 1.5 |
| 20 | Smox | NM_145533 | 2.2 | 3.1 | 5.7 | **2.1** | 1.4 | 0.9 |
| 21 | Kbtbd11 | NM_029116 | 6.2 | 6.9 | 5.6 | **5.2** | 1.7 | 1.6 |
| 22 | Slc35f2 | NM_028060 | 19.3 | 8.1 | 5.4 | **2.7** | 1.9 | 1.4 |
| 23 | Fst | NM_008046 | 2.4 | 4.4 | 5.3 | 1.9 | 1.5 | 1.0 |
| 24 | Espn | NM_207687 | 2.1 | 4.4 | 5.1 | **6.9** | 1.7 | 1.4 |
| 25 | Tnfrsf23 | NM_024290 | 3.2 | 3.3 | 5.1 | **3.5** | 1.5 | 1.1 |
| 26 | Eid2 | NM_198425 | 2.6 | 3.4 | 5.0 | **2.6** | **2.2** | 1.2 |
| 27 | Samd4 | NM_001037221 | 2.4 | 2.3 | 4.0 | 1.2 | 1.2 | 0.9 |
| 28 | Procr | NM_011171 | 2.3 | 4.7 | 4.0 | **3.3** | **2.0** | 1.6 |
| 29 | Usp18 | NM_011909 | 3.7 | 3.4 | 3.9 | **2.4** | 1.3 | 1.4 |
| 30 | Aldh3a2 | NM_007437 | 2.3 | 2.3 | 3.8 | 1.3 | 1.4 | 1.0 |
| 31 | Uap1l1 | NM_001033293 | 3.3 | 3.5 | 3.8 | **7.3** | 1.1 | 1.2 |
| 32 | Lmo2 | NM_008505 | 2.1 | 2.1 | 2.8 | 1.1 | 1.4 | 0.9 |
| 33 | Dnajb11 | NM_026400 | 0.23 | 0.11 | 0.11 | 0.9 | 1.1 | 0.9 |
| 34 | Cadm4 | NM_153112 | 0.32 | 0.25 | 0.05 | 1.1 | 0.9 | 1.0 |
| 35 | Lect1 | NM_010701 | 0.19 | 0.05 | 0.03 | 1.4 | 1.4 | 1.0 |

* - Histone H3K4me3 in genes in bold are statistically significantly different from control

age-matched mice (P ≤ 0.05, fold change ≥2).
