# Supplementary material for: Gene Expression and DNA Methylation Alterations During Non-alcoholic Steatohepatitis-Associated Liver Carcinogenesis
Source: Front Genet. 2019 May 29;10:486. doi: 10.3389/fgene.2019.00486 (PMC6549534; doi:10.3389/fgene.2019.00486)
Supplement: Supplementary file 7 [file Table_4.DOC]

**Supplementary Table 4.** Differentially expressed cytoskeletal-related genes during NASH-associated liver carcinogenesis.

| **NAFL, 6 weeks** | | | | **NASH-fibrosis, 12 weeks** | | | | **HCC, 20 weeks** | | | | |
| --- | --- | --- | --- | --- | --- | --- | --- | --- | --- | --- | --- | --- |
| **#** | **Gene ID** | **Gene name** | **FC*** | **#** | **Gene ID** | **Gene name** | **FC** | **#** | **Gene ID** | **Gene name** | | **FC** |
| 1 | NM_001039000 | *KIF5A* | 3.4 | 1 | NM_019645 | *PKP1* | 13 | 1 | NM_028109 | *TPX2* | 31 | |
| 2 | NM_001042534 | *CAPG* | 3.1 | 2 | NM_177340 | *SYNPO* | 6 | 2 | NM_145150 | *PRC1* | 25 | |
| 3 | XM_006535098 | *SEPT11* | 2.6 | 3 | AK136063 | *SEPT11* | 6 | 3 | NM_009772 | *BUB1* | 24 | |
| 4 | NM_010730 | *ANXA1* | 2.3 | 4 | NM_001039000 | *KIF5A* | 6 | 4 | NM_011121 | *PLK1* | 18 | |
| 5 | NM_023223 | *CDC20* | 2.2 | 5 | NM_001081363 | *CENPF* | 4 | 5 | NM_009264 | *SPRR1A* | 17 | |
| 6 | NM_007864 | *DLG4* | 2.1 | 6 | NM_207687 | *ESPN* | 4 | 6 | NM_001042421 | *KNTC1* | 17 | |
| 7 | NM_207687 | *ESPN* | 2.1 | 7 | NM_001162500 | *PARVG* | 4 | 7 | NM_144553 | *DLGAP5* | 16 | |
|  |  |  |  | 8 | NM_013560 | *HSPB1* | 4 | 8 | NM_001081363 | *CENPF* | 14 | |
|  |  |  |  | 9 | NM_001277875 | *Tpm2* | 4 | 9 | NM_011623 | *TOP2A* | 14 | |
|  |  |  |  | 10 | NM_028109 | *TPX2* | 4 | 10 | NM_001110265 | *TTK* | 13 | |
|  |  |  |  | 11 | NM_019391 | *LSP1* | 4 | 11 | NM_023223 | *CDC20* | 13 | |
|  |  |  |  | 12 | NM_001256002 | *CACNA1C* | 3 | 12 | NM_001012273 | *BIRC5* | 13 | |
|  |  |  |  | 13 | NM_009990 | *CLIP2* | 3 | 13 | NM_001290662 | *KIF2C* | 12 | |
|  |  |  |  | 14 | NM_001042534 | *CAPG* | 3 | 14 | NM_007630 | *CCNB2* | 10 | |
|  |  |  |  | 15 | NM_011623 | *TOP2A* | 3 | 15 | NM_007659 | *CDK1* | 10 | |
|  |  |  |  | 16 | NM_183046 | *KIF20B* | 3 | 16 | NM_011497 | *AURKA* | 10 | |
|  |  |  |  | 17 | NM_145150 | *PRC1* | 3 | 17 | NM_028390 | *ANLN* | 8 | |
|  |  |  |  | 18 | NM_001164567 | *VILL* | 3 | 18 | NM_177340 | *SYNPO* | 7 | |
|  |  |  |  | 19 | NM_011121 | *PLK1* | 3 | 19 | NM_183046 | *KIF20B* | 7 | |
|  |  |  |  | 20 | NM_009772 | *BUB1* | 3 | 20 | NM_009450 | *TUBB2A* | 7 | |
|  |  |  |  | 21 | NM_018873 | *SRCIN1* | 3 | 21 | NM_207687 | *ESPN* | 5 | |
|  |  |  |  | 22 | NM_001278269 | *FYB* | 3 | 22 | NM_008634 | *MAP1B* | 5 | |
|  |  |  |  | 23 | NM_001289722 | *WIPF1* | 2 | 23 | NM_008446 | *KIF4A* | 5 | |
|  |  |  |  | 24 | NM_027395 | *BASP1* | 2 | 24 | NM_010730 | *ANXA1* | 5 | |
|  |  |  |  | 25 | NM_030249 | *CTTNBP2NL* | 2 | 25 | NM_010620 | *KIF15* | 4 | |
|  |  |  |  | 26 | NM_019682 | *DYNLL1* | 2 | 26 | AK032942 | *Tpm1* | 4 | |
|  |  |  |  | 27 | NM_001001491 | *Tpm4* | 2 | 27 | NM_016692 | *INCENP* | 4 | |
|  |  |  |  | 28 | NM_023223 | *CDC20* | 2 | 28 | NM_011701 | *VIM* | 4 | |
|  |  |  |  | 29 | NM_010629 | *KIFAP3* | 2 | 29 | NM_019391 | *LSP1* | 4 | |
|  |  |  |  | 30 | NM_008697 | *NIN* | 2 | 30 | NM_010615 | *KIF11* | 4 | |
|  |  |  |  | 31 | NM_031998 | *CEP41* | 2 | 31 | NM_009609 | *ACTG1* | 3 | |
|  |  |  |  | 32 | NM_009406 | *TNNI3* | 2 | 32 | NM_001042534 | *CAPG* | 3 | |
|  |  |  |  | 33 | NM_008538 | *Marcks* | 2 | 33 | NM_017464 | *NEDD9* | 3 | |
|  |  |  |  | 34 | NM_144800 | *MTSS1* | 0.5 | 34 | NM_001256002 | *CACNA1C* | 3 | |
|  |  |  |  | 35 | NM_012030 | *SLC9A3R1* | 0.5 | 35 | NM_025995 | *FBXO5* | 3 | |
|  |  |  |  | 36 | NM_001042542 | *AKAP4* | 0.4 | 36 | NM_009990 | *CLIP2* | 3 | |
|  |  |  |  | 37 | NM_001080966 | *AURKC* | 0.3 | 37 | NM_010892 | *NEK2* | 3 | |
|  |  |  |  |  |  |  |  | 38 | NM_024245 | *KIF23* | 3 | |
|  |  |  |  |  |  |  |  | 39 | NM_001001491 | *Tpm4* | 3 | |
|  |  |  |  |  |  |  |  | 40 | AK136063 | *SEPT11* | 3 | |
|  |  |  |  |  |  |  |  | 41 | NM_018855 | *GAS8* | 3 | |
|  |  |  |  |  |  |  |  | 42 | NM_001290421 | *FLNA* | 3 | |
|  |  |  |  |  |  |  |  | 43 | NM_146009 | *CEP290* | 3 | |
|  |  |  |  |  |  |  |  | 44 | NM_001301374 | *CORO1A* | 2 | |
|  |  |  |  |  |  |  |  | 45 | NM_011655 | *TUBB5* | 2 | |
|  |  |  |  |  |  |  |  | 46 | NM_009499 | *VASP* | 2 | |
|  |  |  |  |  |  |  |  | 47 | NM_134080 | *FLNB* | 2 | |
|  |  |  |  |  |  |  |  | 48 | NM_008538 | *Marcks* | 2 | |
|  |  |  |  |  |  |  |  | 49 | NM_030249 | *CTTNBP2NL* | 2 | |
|  |  |  |  |  |  |  |  | 50 | NM_010629 | *KIFAP3* | 2 | |
|  |  |  |  |  |  |  |  | 51 | NM_144800 | *MTSS1* | 0.5 | |
|  |  |  |  |  |  |  |  | 52 | NM_012030 | *SLC9A3R1* | 0.4 | |
|  |  |  |  |  |  |  |  | 53 | NM_178633 | *KLHL2* | 0.4 | |
|  |  |  |  |  |  |  |  | 54 | NM_175138 | *DNAI1* | 0.4 | |
|  |  |  |  |  |  |  |  | 55 | NM_007462 | *APC* | 0.3 | |
|  |  |  |  |  |  |  |  | 56 | NM_177678 | *ABLIM2* | 0.3 | |
|  |  |  |  |  |  |  |  | 57 | NM_001164171 | *MYH6* | 0.3 | |
|  |  |  |  |  |  |  |  | 58 | NM_001042542 | *AKAP4* | 0.2 | |
|  |  |  |  |  |  |  |  | 59 | NM_009864 | *CDH1* | 0.2 | |
|  |  |  |  |  |  |  |  | 60 | NM_009797 | *CAPZA1* | 0.2 | |
|  |  |  |  |  |  |  |  | 61 | NM_001289659 | *UPP2* | 0.1 | |
|  |  |  |  |  |  |  |  | 62 | NM_138313 | *BMF* | 0.1 | |
|  |  |  |  |  |  |  |  | 63 | AK138560 | *SPTBN2* | 0.1 | |

* - FC – Fold change.
