# Supplementary material for: Gene Expression and DNA Methylation Alterations During Non-alcoholic Steatohepatitis-Associated Liver Carcinogenesis
Source: Front Genet. 2019 May 29;10:486. doi: 10.3389/fgene.2019.00486 (PMC6549534; doi:10.3389/fgene.2019.00486)
Supplement: Supplementary file 8 [file Table_5.DOC]

**Supplementary Table 5.** Gene expression and gene-specific methylation of epigenetically regulated genes uniquely expressed in full-fledged HCC during NASH-associated liver carcinogenesis.

| **#** | **Gene name** | **Gene ID** | **Gene expression, fold change** | | | **Gene methylation, % of input** | | | | | |
| --- | --- | --- | --- | --- | --- | --- | --- | --- | --- | --- | --- |
|  |  |  | **6 weeks** | **12 weeks** | **20 weeks** | **NAFL, 6 weeks** | | **NASH-fibrosis, 12 weeks** | | **HCC, 20 weeks** | |
|  |  |  |  |  |  | **Control** | **STAM** | **Control** | **STAM** | **Control** | **STAM** |
| 1 | Slc25a37 | NM_026331 | NS | NS | 34.7 | 6.7 | 5.6 | 4.9 | 5.4 | 7.9 | **4.5*** |
| 2 | Nuf2 | NM_023284 | NS | NS | 19.4 | 5.2 | 4.8 | 4.8 | 5.2 | 10.1 | **5.0** |
| 3 | Ccnb2 | NM_007630 | NS | NS | 10.1 | 8.8 | 9.8 | 7.8 | 6.6 | 11.0 | **6.4** |
| 4 | Mxd3 | NM_016662 | NS | NS | 7.8 | 7.0 | 8.3 | 6.8 | 7.8 | 7.1 | 5.1 |
| 5 | Hells | NM_008234 | NS | NS | 5.8 | 8.0 | 6.9 | 5.6 | 5.3 | 7.2 | **4.1** |
| 6 | Slc7a1 | NM_007513 | NS | NS | 4.0 | 12.7 | 12.2 | 9.2 | 8.3 | 10.8 | **6.4** |
| 7 | Pdgfb | NM_011057 | NS | NS | 3.3 | 6.6 | 8.1 | 6.9 | 5.3 | 10.2 | **5.2** |
| 8 | Fbxo5 | NM_025995 | NS | NS | 3.2 | 6.0 | 5.7 | 5.4 | 3.9 | 8.3 | **5.9** |
| 9 | Ppm1h | NM_176919 | NS | NS | 2.9 | 11.9 | 9.6 | 11.0 | 12.0 | 14.2 | **6.2** |
| 10 | Dusp10 | NM_022019 | NS | NS | 2.9 | 6.3 | 6.5 | 5.8 | 4.9 | 10.5 | **5.3** |
| 11 | Ints2 | NM_027421 | NS | NS | 2.8 | 6.6 | 7.6 | 5.3 | 5.9 | 6.7 | **3.8** |
| 12 | Dll4 | NM_019454 | NS | NS | 2.6 | 7.5 | 8.0 | 9.2 | 9.4 | 9.3 | **5.6** |
| 13 | Ggta1 | NM_010283 | NS | NS | 2.5 | 10.2 | 11.2 | 8.3 | 7.5 | 10.3 | **6.0** |
| 14 | Nfkb2 | NM_019408 | NS | NS | 2.4 | 4.4 | 5.2 | 5.6 | 6.5 | 7.4 | 6.2 |
| 15 | Mkrn1^#^ | NM_018810 | NS | NS | 2.4 | 89.6 | 94.5 | 92.1 | 90.5 | 95.3 | **75.5** |
| 16 | Nudt18 | NM_153136 | NS | NS | 2.4 | 6.7 | 6.1 | 6.7 | 5.9 | 10.5 | **6.1** |
| 17 | Vasp | NM_009499 | NS | NS | 2.2 | 5.9 | 4.6 | 4.3 | 4.0 | 4.5 | **3.0** |
| 18 | Sqstm1 | NM_011018 | NS | NS | 2.2 | 6.1 | 4.0 | 3.8 | 3.4 | 7.0 | **4.4** |
| 19 | Cmtm6 | NM_026036 | NS | NS | 0.5 | 6.8 | 7.8 | 5.4 | 6.2 | 7.3 | **12.6** |
| 20 | Bcat2 | NM_009737 | NS | NS | 0.5 | 58.7 | 67.0 | 61.9 | 59.7 | 55.7 | **85.0** |
| 21 | Rbpms2 | NM_028030 | NS | NS | 0.5 | 8.0 | 6.9 | 6.5 | 5.9 | 7.7 | **12.7** |
| 22 | Sash1 | NM_175155 | NS | NS | 0.5 | 8.4 | 7.9 | 6.7 | 6.1 | 8.1 | **13.6** |
| 23 | Pim3 | NM_145478 | NS | NS | 0.4 | 6.3 | 7.0 | 4.5 | 4.0 | 7.2 | **15.2** |
| 24 | Rgs6 | NM_015812 | NS | NS | 0.4 | 9.8 | 13.9 | 12.8 | 9.6 | 12.8 | **25.2** |
| 25 | Bcl9l | NM_030256 | NS | NS | 0.3 | 39.9 | 43.1 | 38.2 | 42.6 | 34.6 | **49.0** |
| 26 | Fbxo31 | NM_133765 | NS | NS | 0.3 | 6.6 | 6.1 | 5.2 | 5.3 | 7.4 | **14.7** |
| 27 | Smc1b | NM_080470 | NS | NS | 0.3 | 44.9 | 45.1 | 27.9 | 31.4 | 25.9 | **42.9** |
| 28 | Mier1 | NM_001039081 | NS | NS | 0.3 | 7.1 | 8.7 | 8.1 | 7.8 | 13.8 | 17.2 |
| 29 | Lypd3 | NM_133743 | NS | NS | 0.2 | 34.2 | 37.4 | 24.1 | 26.6 | 18.2 | **32.6** |
| 30 | Gdf10 | NM_145741 | NS | NS | 0.2 | 13.7 | 13.3 | 10.1 | 10.1 | 10.3 | **18.4** |
| 31 | Fryl | NM_028194 | NS | NS | 0.2 | 5.3 | 7.1 | 6.9 | 6.0 | 8.0 | **14.8** |
| 32 | Lhx2 | NM_010710 | NS | NS | 0.2 | 6.1 | 4.9 | 5.5 | 4.9 | 6.4 | **9.8** |
| 33 | Klhl32 | NM_001033531 | NS | NS | 0.1 | 7.7 | 8.5 | 10.2 | 9.9 | 18.2 | **32.2** |
| 34 | Ndn | NM_010882 | NS | NS | 0.1 | 32.0 | 33.7 | 31.2 | 31.4 | 33.1 | **52.8** |

* - methylation of genes in bold are statistically significantly different from control age-matched mice (P ≤ 0.05); NS – not significant; ^#^ - underlined are genes presented in Figure 4.
